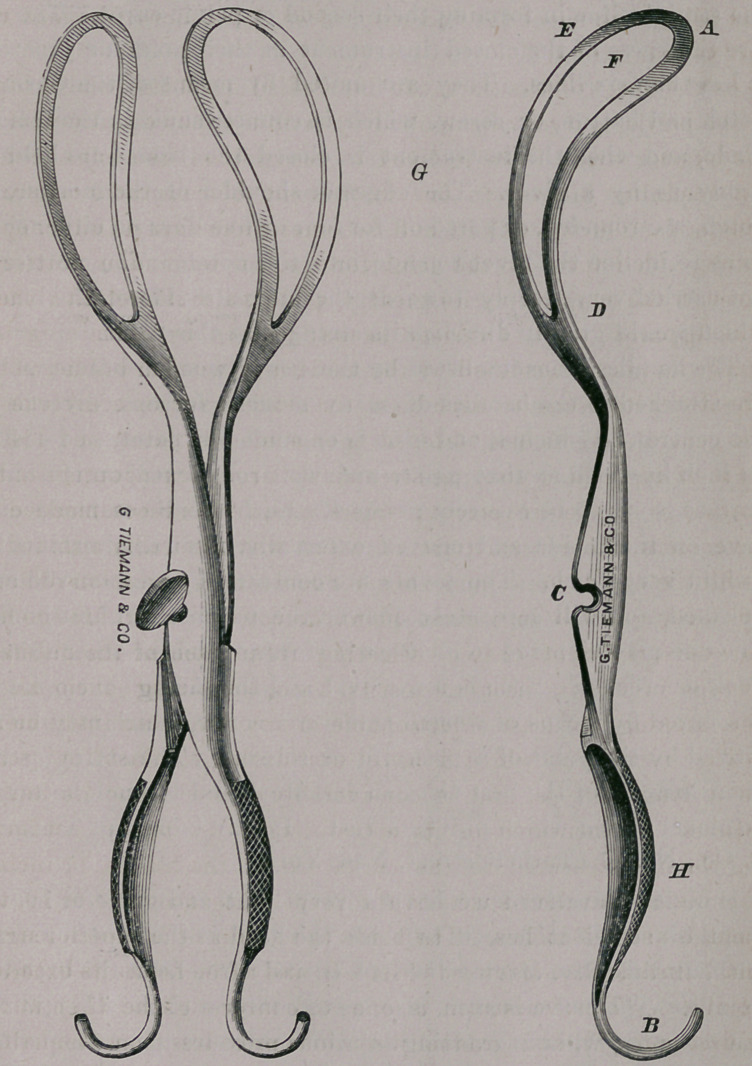# The Obstetrical Forceps

**Published:** 1878-06

**Authors:** James P. White


ART. II.—*The Obstetrical Forceps*—by Prof. James P. White, M. D. Presented before the Buffalo Medical Association April 2d, 1878. Reported by Joseph Fowler, M. D., *Secretary*.

The first to mention the forceps was Avicenna, in the eleventh century, but during the succeeding five or six hundred years it seems to have been entirely forgotten until a member of the family of the Chamberlens, in the first half of the seventeenth century, invented and secretly used an instrument with which he claimed to be able to deliver women more speedily and with less danger than if the completion of labor was left to nature. The secret remained with the Chamberlens, who refused to divulge their appliances, and it was only during the first half of the eighteenth century, after Chapman and Gifford had introduced a straight instrument, that it was brought into general acquaintance. It is, therefore, a comparatively modern invention, and many modifications have been brought forward which greatly increase its usefulness. The form of the forceps employed differs greatly; in England a short, straight instrument is chiefly used, while on the continent we find a long one with the pelvic curve. In America both are used, as the practitioner chanced to adopt as his text-book and guide, a French or English author.

The value of the forceps depends upon their applicability, and in this respect the long are much to be preferred to the short; the latter can be employed only at the inferior straight, whereas the former can be made available equally well at the brim of the pelvis. It is due to this difference, in part, at least, that statistics concerning the frequency of the use of the forceps vary so much. While in France and Germany they are resorted to by some as often as one in every seven, and, by those obstetricians who use them least, as often as once in "250" labors; in England and Ireland only one in six or seven hundred has until recently been thought suitable for their application. Thus we find Dr. Seibold, of Berlin, who uses a long instrument, according to the valuable tables furnished by the American edition of Churchill's Midwifery, had recourse to forceps once in every seven cases—and *craniotomy* only once in 2093 cases. Dr. Collins, of Dublin, who recommends the short forceps, employed it only once in 617 labors, and resorted to the graver operation of *perforation* once in 141 cases, being nearly 4½ times as often as he used the forceps. These men were among the very first practitioners in their respective countries, and yet we find the celebrated Irish accoucheur resorted to craniotomy more than 14 times as often as the no less distinguished continental physician, whilst the latter delivered by forceps 88 times as often as the former. Nor are these by any means rare examples. That the highest proportional frequency may not claim imitation we admit, but is it not apparent that there may be danger of falling into the opposite extreme, and that hyper-caution and delay may beget the necessity, often, for a more frequent resort to craniotomy ?

As the long forceps only, can be applied when the head is high in the pelvis, and may be apolied by skillful hands equally well at the inferior strait, I am inclined to recommend its exclusive use. By confining himself to one instrument, the operator acquires greater familiarity with it, and becomes more expert in its application. The form of the long forceps, as we find it scattered through the country, varies greatly, as does also its weight ; there is no apparent want of variety, as may be seen by examining the numerous plates furnished by the modern obstetrical publications. The difficulty seems to have been, that each inventor conscious of the general defects, has fastened upon some one point, and losing sight of everything else, has strenuously urged the adoption of his fancied or real improvement ; others, again, have recommended an instrument, the general form of which was admirably adapted to fulfill the end in view, and then rendered its application difficult by leaving some important point defective. Prof. Hodge has pursued a different course, selecting from each of the different forceps in use its peculiar merits, then combining them all in one, making the least objectionable of any American instrument.

The instrument which I have used during the last few years is a long forceps, and is considerably curved upon its lateral aspects. It measures in its entire length (*a* to *b*), conforming the line measured to the curvature of the blades, 17 inches. The blades and their shafts to the pivot being about 9½ or 10, the handles about 7 inches. The blade (*a* to *d*) is 6½ inches in length, and 7 lines at its narrowest point (*d*), and 2⅛ inches in its broadest point (*e*). The fenestrum is one and three-eighths (1⅜) at the widest part (*f*), and gradually diminishes to less than one-half of an inch at the heel. The inner or fenestral margin of the blades is ground down so as not to exceed one-sixteenth of an inch in thickness, the width (*e* to *f*) being scarcely $5\frac{1}{2}$ lines, and not exceeding one line in thickness at its periphery (*e*), being considerably thicker in the centre (midway between *e* and *f*).

The shaft of the blade is scalloped out considerably toward the pivot, upon its inner surface, beyond the termination of the fenestrum, thus diminishing weight without lessening the strength.

The points of the blades when the instrument is closed (*a*) are but 8 or 9 lines apart, and at the widest point (*g*) they are 2 inches and 9 lines apart, on the upper or concave surface; whilst on the lower or convex surface, they are slightly more expanded. The shafts of the blades (from *d* to *c*) approach each other rapidly, but not abruptly.

The blades at the centre of their points deviate 3½ inches from the straight line in forming their second or pelvic curve. The entire thickness of the closed instrument at their point of junction is less than six lines. They are united by means of the German notch and button, or screw, which is counter-sunk in the female blade, and when the instrument is closed the blades are held as firmly as by a pivot. The edges or shoulder of the mortise, or notch, are rounded, or pared off for four or five lines on either side, so as to incline the pivot to slide into the notch. The mortise is not carried very deeply towards the opposite side of the blade, which would greatly diminish its strength at this point.

The handles, unencumbered by the heavy wooden beams which are attached to the handles of many modern forceps, diverge in the centre to 1¾ inches, and each is expanded or flattened to 1⅛ of an inch in width at that point, and well roughened on the outer surface, so as to be securely grasped. Each handle is made concave on the inside and convex externally, thus diminishing its weight very much. The points are contracted again, curved and polished, and will separately answer the purpose of blunt hooks. The one may be made to inclose a perforator, and the other a sharp hook or crochet. Each is made oval, and the sheath enveloping it is secured by means of a small transverse screw, which may be removed by the point of a penknife or scissors. The entire instrument is made of the best German cast-steel, and is much better to be nickel plated which prevents rust. It may be had of Tiemann & Co., No. 67 Chatham street, New York.

Here it is perceived we have a very light and graceful instrument of sufficient length to seize the head at the superior strait without difficulty, leaving the lock entirely free from the external organs. The curve is such, also, as to conform to the direction of the passages, without exerting injurious pressure upon the perineum. The shafts of the blade approximate so as not to distend the vulva before the descent of the head. They incline, however, so gradually as not to diminish their power, as is the case with the instrument of Dr. Hodge.

It will be found that the concavity of the fenestrum, beveling off the inner edges of the blades, will render it better adapted to fit accurately the parietal protuberances, and prevent those salient points from being injured or indented by the sharp angles usually found on the inner border of the fenestrum. Moreover, this is the widest part of the fœtal head and the surface to which the fenestrum is ordinarily applied, and if this margin of each blade be two or two and a-half lines in thickness, as is the case in many instruments, the amount of compression of the head must be three lines more in consequence of unnecessary thickness. One of the difficulties in application consists in uniting the blades. In the instrument represented this end is greatly facilitated, slightly lessening the weight at the same time, by cutting away the abrupt shoulders to the mortice, into which the screw easily glides, whenever it gets within these inclined planes. Again, whoever has been compelled to hold on to well polished round steel handles will readily appreciate the comfort, as well as sense of security, which a roughened and expanded surface must afford. The length of the handle may be increased and bent so as to form a blunt hook, and a very good perforator may be inserted into the extremity of one handle and a sharp hook into the other, which will answer very well if the work of destruction become unavoidable.

The weight of the different forceps most frequently found in use, is as follows:—

| | | | |
|---|---|---|---|
| White's, | 13 ounces, | Eliots, | 19¼ ounces, |
| Hodge's | 17½ " | Suer, Paris, | 23 " |
| Bedford's, | 18½ " | Seibold, Berlin, | 27 " |

The last four have their weight greatly increased by the heavy wooden handles, which are entirely dispensed with in the first instrument. Some manufacturers, in their endeavor to make a light and graceful instrument have sacrificed strength to beauty, and rendered them of little value from the liability of the blades to spring apart and dilate, slipping from their hold when force is applied. More compression can be made with White's than with Hodge's forceps, although they are $4\frac{1}{2}$ ounces lighter. This is due to the gradual inclination of the blades to each other.

In 1851, while in Paris, two instruments were constructed by Cherriere, the French instrument maker, in the manner just described, being exact copies of a pair of forceps which I carried with me. One of these was presented to Paul Dubois, who assured me at a subsequent visit in 1866 that he had used it ever since with great satisfaction. Exact patterns of White's forceps are now largely sold in Paris and New York as Dubois' instrument.

Of the complex and complicated instruments introduced by M. Tarnier, of France, and of the still more modern one by Dr. M'-Ferran, in which by means of a joint, motion of the blades is allowed, little need be said. They present no benefits over the ordinary forceps, and will be found more difficult of application and less certain in use; this is as true of most of the changes proposed. The light, strong, well-constructed, long, curved forceps in the hands of a skillful practitioner are the safest mode of instrumental assistance.

The prejudice of British and American practitioners to the more frequent use of this most beneficent of all obstetrical instruments is rapidly giving place to a more intelligent appreciation of its powers and utility. Barnes, Playfair, Aveling and other celebrated English obstetricians all advise their more frequent employment, and Dr. Edis gives as one of his conclusions, in an article on the forceps recently read before the obstetrical society that "the use of forceps, at least as often as in one case in ten, is desirable." This view was concurred in by nearly all the noted obstetricians present, and showed that a change amounting to a revolution has taken place in the opinion and practice of London practitioners.

The early application of the forceps diminishes the danger of septicæmia, sloughing, stricture and laceration, since these depend more upon the duration of the labor than the severity. Some of the indications warranting their employment are, inertia of the uterus, slow descent of the head through the pelvis, a pelvic diameter below three and one-half ($3\frac{1}{2}$) inches, delay for any cause, rupture of the uterus where presenting part can be seized, and in breach presentations where the danger to the child is very great.

Traction should always be made in the direction of the axis of the straight in which the head is placed and in imitation of nature, periodically.

Common sense should be used in determining how and when to apply the forceps, and in using heavy ones the danger of wounding the head of the child and maternal parts must be borne in mind. It is better to err by interfering, perhaps, a little too soon, rather than sit supinely by, while the strength and vital force of the patient ebbs away.

## Figures and Tables

**Figure f1:**